# Occurrence of Microplastics in Harbour Seals (*Phoca vitulina*) and Grey Seals (*Halichoerus grypus*) from German Waters

**DOI:** 10.3390/ani12050551

**Published:** 2022-02-23

**Authors:** Carolin Philipp, Bianca Unger, Ursula Siebert

**Affiliations:** Institute for Terrestrial and Aquatic Wildlife Research, University of Veterinary Medicine Hannover, Foundation, Werftstraße 6, 25761 Buesum, Germany; bianca.unger@tiho-hannover.de

**Keywords:** marine mammals, Phocidae, North Sea, Baltic Sea, gastrointestinal tract, intestine, stomach, fibres, fragments

## Abstract

**Simple Summary:**

It is well known that the occurrence of microplastics (MP) is ubiquitous in both water compartments and in biota. Nevertheless, knowledge on the microplastic exposure of marine mammals is scarce. This study gives first evidence of microplastic burden in seal species inhabiting German waters of the North Sea and Baltic Sea. Accordingly, 63 gastrointestinal tracts (GIT) of harbour seal (*Phoca vitulina*) and grey seal (*Halichoerus grypus*) carcasses were analysed. This investigation is urgently needed since information on microplastic burden in grey seals, especially from the Baltic Sea, are still lacking. This information is particularly important as it allows a direct comparison of this species and its sympatric species, the harbour seal, from different waters. A slightly higher incidence of microplastics (>100 µm, here categorized as fibres and fragments) was found in specimen found in the Baltic Sea. Moreover, higher microplastic loads were detected in stomachs compared to the intestinal samples, with first evidence of fragment retainment within the GIT. Nevertheless, an accumulation of microplastics in marine mammals could not be excluded for all categories since the egestion of those subjects is documented and no significant differences in life history parameters such as sex and age, nor parasite infestation were revealed.

**Abstract:**

The level of knowledge on microplastic exposure in marine mammals is limited by the access to dead and alive individuals. Focusing on the Northeast Atlantic area, some studies already confirmed the microplastic presence in free-ranging marine mammals, such as harbour porpoises or harbour seals inhabiting the North Sea (NS). In contrast, knowledge on the exposure to grey seals and particularly on specimen inhabiting the Baltic Sea (BS) are scarce. This study examined 63 gastrointestinal tracts (GIT) of harbour seals and grey seals originating from German waters (NS and BS) found between 2014 and 2019. Besides the documentation of microplastic findings, this study is dealing with life history and health parameters, attempting to identify correlations with microplastic presence. This study confirmed beside the presence, the egestion of microplastics (>100 µm; MPs) in the examined seals, without correlations in parasite infestations or inflammation responses. 540 suspected MPs were identified in 62 intestinal samples (42% fibres, 58% fragments), and 228 MPs in seven stomachs (28% fibres, 72% fragments). In accordance, first evidence of the retainment of fragments in the GIT were given. However, no significant difference in MP occurrence was indicated for different sex or age groups.

## 1. Introduction

The awareness of marine litter presence and its impacts on marine biota increased in recent years [1]. In accordance, the number of studies dealing with the occurrence and its effects has also increased. Several studies already revealed the risk of entanglement and ingestion in or of marine litter also for marine mammals occurring in the North Sea (NS) and Baltic Sea (BS) [2,3,4]. Moreover, the impacts of ingested microplastic (MP) on invertebrates are described in numerous laboratory studies [5,6,7]. However, studies on MPs and their occurrence in free-ranging marine mammals as well as their impact are still lacking, since laboratory studies on marine mammals are ethically not justifiable, and thus not viable [8]. In addition, predator species such as seals generally take MP up while feeding on burdened prey species [9]. Thus, monitoring the marine environment (e.g., water or sediment) is not sufficient to determine the burden in seals, for example. Moreover, different feeding behaviour or different feeding habitats can additionally result in varying MP burden in marine mammals [10,11,12,13,14].

First evidence of the MP burden in the main regularly occurring top predator species are given in studies focusing on marine mammals found along the German and Dutch coastline; two studies investigated harbour porpoises [14,15] and another pilot study examined a small sample size of harbour seals and grey seals [16]. The aforementioned studies are comparable with examinations on porpoise and seal carcasses or scat samples which were conducted around the coastline of Great Britain and Ireland [17,18,19], since marine mammals are highly mobile within the NS and adjacent waters [13,20]. Knowledge on the MP occurrence in marine mammals, especially on seal species inhabiting the BS, which is known to be impacted by contaminants [21], is still scarce. Up to the present date, only one study investigated intestinal specimens of harbour porpoises found along the German coastline of the BS, and thus provided first findings in marine top predators in this sea [14]. Those three latter mentioned species (harbour seal, grey seal and harbour porpoise) are the most common marine mammals in the NS and the BS [20,22], thus information on the MP burden needs to be gained. Moreover, to investigate top predator species of the marine environment for MP occurrence completes the knowledge of MP transfer within the whole food web, since effects are mainly studied for invertebrates and not for mammals [23].

Furthermore, Germany as part of the EU is obliged to monitor and inform on the burden caused by anthropogenic impacts on their waters (e.g., MSFD) [24]. Therefore, the results presented here are highly valuable to serve this obligation.

To fill the gap of this lacking information on MP occurrence in seals, this study investigated intestinal and stomach samples of harbour seals (*Phoca vitulina*) and grey seals (*Halichoerus grypus*) found along the German coastline. This allows for an insight into the MP burden of those species occurring in both, the German NS and BS. Moreover, this study is taking information on the age and sex of the investigated individuals into consideration, as well as records of gastrointestinal lesions and parasite infestations to reveal possible relations. The evaluation of lesions such as inflammatory responses and MP occurrence was focused, because a risk of damage in the GIT tissue by those small particles and consequently an inflammation cannot be totally excluded, since it is already well documented in invertebrates and mice [25,26].

Moreover, the inclusion of health parameters aims to identify conceivable relations triggered by a possible MP burden in those specimens. Similar studies were already performed with different marine mammal species in the Northeast Atlantic region [14,17,19]. However, to date and to best knowledge of the authors, no comparable study was performed for seals occurring in the BS. In accordance, this study gives first evidence of the MP burden in seals from the BS for the first time. To set the outcomes of both species and both seas to each other and to already published studies from the north-eastern Atlantic [14,17,19], the investigated specimens of the NS and BS were processed in the same manner, thus making the comparison of the results highly reliable.

## 2. Materials and Methods

### 2.1. Sample Collection & Sample Treatment

Since 1990, the Institute for Terrestrial and Aquatic Wildlife Research (ITAW) regularly conducts necropsies of stranded, bycaught or mercy-killed marine mammals found at the coasts of the federal state of Schleswig-Holstein in Germany [27,28,29]. Every intestinal sample was taken of 8 to 10 cm from the hindgut (*Intestinum rectum*) and was selected in case faeces were present and the gastrointestinal tract (GIT) was undamaged [16]. The intestinal samples were collected with metal scalpels and tweezers, immediately after opening the abdominal cavity of the carcass during those necropsies. Afterwards, the samples were stored in cleaned and disinfected glass jars at −20 °C until further treatment. All processing steps were conducted in a closed acrylic box, with openings for the hands, wearing cotton gloves above nitrile ones. The intestinal samples were rinsed with MilliQ water (Millipore), placed in a double-layer nylon fabric with different mesh sizes (inner fabric: 300 µm; outer fabric: 100 µm) and opened within it. Afterwards, the fabric was folded and sewed with a sewing machine. Subsequently, this self-sewed washing sachets (including the sample) are washed in a conventional washing machine at 60 °C without a spinning cycle, adding enzyme detergents. This cleansing step reduces organic matter and restrains hard parts such as sand grains or prey remains (e.g., fish bones and otoliths), but also potential MP particles. For MP isolation, first a density separation with saturated sodium chloride solution (NaCl) was conducted before applying a Nile Red staining technique which visualize potential MP particles using fluorescence microscopy [30]. A detailed description of the sample handling, treatment and particle selection is given in Philipp et al. [16].

This study also investigates seven belonging stomachs of harbour seals and a grey seal in addition to the intestinal samples, all gathered in 2019. Those stomachs were taken into account to extend the analysis of the GITs focusing on MPs and enlarge the sample collection in addition to the priorly collected intestinal samples. In six out of the seven cases, both the intestinal sample and the stomach were examined. For the remaining stomach investigated, no intestinal sample could be analysed due to lacking faeces. The stomachs were completely removed from the carcasses, ligated with draw strings at the cardia and the pylorus to avoid any loss of the stomach content. Afterwards, the collected stomach was transported covered in aluminium foil to the working environment, the closed acrylic box, for further processing: The stomach was rinsed with MilliQ water from the outside and the drawstrings were removed prior opening it above the nylon fabrics. The suture, cleansing and all other subsequent steps were conducted after the same protocol, as it was used for the intestinal samples described above and after Philipp et al. [16].

To select only specimen which already preyed independently, ensuring that an MP uptake is considerable, samples of adults and one year old individuals were primarily chosen. Moreover, individuals classified as under a year were chosen if their stomach was filled with prey remains. The further age determination was conducted according to the weight and length of each carcass [27] together with counting of the growth layer groups in the dentine and cementum of the teeth of the individuals [31].

Next to those categories, the samples were selected in dependence on the origin sea and the species to enable a comparison on similar sample size. In total, samples of 62 intestinal samples were investigated from harbour seals and grey seals of NS and BS between 2014 and 2019. This includes also one carcass of a female grey seal which was found on the Danish coastline, since this individual was tagged in German waters by ITAW employees. Moreover, the sample size includes also an individual whereof the origin sea (NS or BS) is unknown. Thus, this individual is excluded from the comparison between the two seas. Those specimens were not generally excluded out of the whole investigation, since this study focused on covering the same time span for both waters (six years) as well as investigating the approximately same number of samples for NS and BS, which were limited by the presence of carcasses. Thus, those two exceptional seals were also considered, next to the individual whereof only the stomach was investigated.

### 2.2. Assessment of Suspected Microplastics

Found MPs in the size range of 100–5000 µm were photographed, measured in size, and categorized as either fragments or fibres. In the following, the term “particle” includes both fragments and fibres. Fibres are particles characterised by a maximal width of 8–23 µm, an elongated shape and a measured length of at least the triple size of the maximal width. All particles not meeting these criteria were classified as fragments. The focused size range of 100–5000 µm was determined by the used mesh size of 100 µm for the washing process and a unified definition of MP [32].

The aim of this study was to evaluate the occurrence of suspected MP and its impacts rather than identify polymer composition. For identifying those MPs under fluorescence light, the following attributes were applied: biogenic look, the fluorescence appearance, and property of the particle (fragmented, melted, textural appearance). For a detailed overview of all categories and a catalogue of examples, see Philipp et al. [16].

### 2.3. Prevention of Secondary Contamination

The whole processing of the samples was conducted in closed acrylic boxes, which are cleaned with MilliQ water and ethanol (70%) prior and after each working step. To monitor the risk of secondary contamination, 27 procedural blanks (including ten procedural blank samples and 17 blanks of the working environment) were considered for estimating the potential risk of secondary contamination. The procedural blank filters include filtrations of the used MilliQ water, NaCl solution and the processed washing sachets which were treated in the same manner than those filled with samples. Whereas the blank filters out of the working environment present the contamination risk from the necropsy room (sample taking) and within the closed acrylic box (sample processing). Those blanks run along with the samples and were handled after the same applied protocol (16). The use of blanks is essential to judge the contamination during sample handling and allow for a most precise evaluation of the actual burden of the animals by avoiding overestimation of the particles in the sample.

The mean values of each procedural blank category were calculated and subsequently summed up. Accordingly, two fibres and seven fragments were determined as secondary contamination per sample and were subtracted from each intestinal or stomach specimen to avoid an overestimation of MPs. Lost fibres of the used nylon fabric were easily determined using fluorescence microscopy, since its unique fibre pattern is identifiable (16). As consequence, those fibres could easily be excluded in the data evaluation.

### 2.4. Pathological Investigations in the GIT

Pathological lesions of the GIT lesions such as inflammations or parasitic infestations were determined as part of necropsies and histopathological investigations and recorded in an internal health database. For inflammatory lesions, e.g., gastritis and enteritis, character, severity (none, mild, moderate or severe), distribution and age of the lesions were determined by veterinarians and pathologists after highly established protocols for the whole GIT [27,28,29,33,34,35] of the investigated specimen. These pathological findings were used to quantify a potential correlation between those effects in the stomach and intestinal tissue, and MP occurrence for the first time in seals.

### 2.5. Statistical Analysis

The statistical analysis was performed in R Version 4.0.2 [36]. The amount of found MP particles are indicated as mean values and standard deviation (M ± SD), thus the findings can be compared between different groups within this study (e.g., location and life history parameter) or with prior investigations (e.g., other species and locations). To evaluate potential correlations (e.g., parasite infestation and MP presence) the package ‘psych’ was used to perform Pearson’s correlation (*p* < 0.05) [37], besides the application of a Kruskal–Wallis Rank Sum Test [38] to evaluate the hypothesis of a correlation between parasite infestation and MP occurrence, and an unpaired t.test (comparison of seas and the species). To further analyse the samples of correlation and to determine important parameter (sex and age, parasitic infestation, and lesions) focusing on the MP burden, the data were applied in one-sided and two-sided ANOVA tests (*p* < 0.001). All graphs were created with the package ‘ggplot2’ [39]. Based on the small sample size of investigated stomachs, only the intestinal samples were considered for observing differences in species, seas, sexes or ages.

## 3. Results

### 3.1. Comparison of North & Baltic Seas

In seals from the NS the MP findings range between zero and 43 particles per intestinal samples (*n*_intestines_ = 39). A total of 326 MPs were identified in those specimen (fibres: 41.72%, fragments: 58.28%). In the intestinal samples of seals found in the BS (*n*_intestines_ = 22), 209 MPs were identified in total. The findings differed between zero and 48 particles per individual. The share of fibres and fragments is similar in individuals found in the BS (fibres: 41.63%, fragments: 58.37%). In seven seals originating from the NS and two individuals from the BS no MP were present, after subtraction of the considered secondary contamination. Unfortunately, no stomach samples were available for these nine examined individuals, since the intestinal samples were collected prior 2019 and no stomachs were taken into account for MP investigations at this time.

Five examined stomachs of harbour seals from the NS showed a MP load from 16 to 53 MPs per sample. The two investigated individuals from the BS both showed the presence of MPs in the stomachs (harbour seal: 47 MPs; grey seal: 37 MPs). In general, the MP occurrence in the stomach was in five out of six related cases higher when compared to the intestinal samples (Table 1). No significant differences in the MP burden between those two species from the two different seas were identified (*p*-value = 0.78). Nevertheless, a slightly larger number of MPs was determined in intestinal samples of individuals from the BS (BS: M ± SD = 9.45 ± 16.92; NS: M ± SD = 8.29 ± 11.62).

### 3.2. Comparison of Species

**Figure 1:**
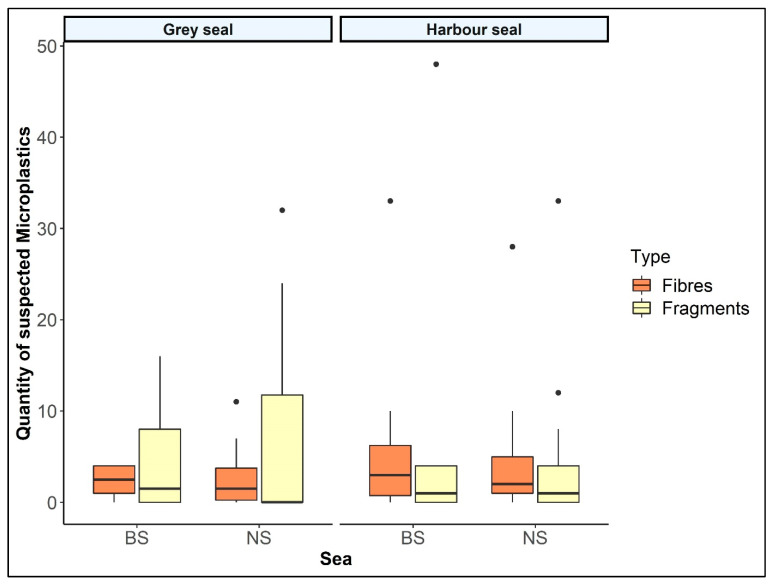
Quantity of suspected microplastics in intestinal samples of seals from German waters separated by origin sea (North Sea: NS; Baltic Sea: BS) and species (*n*_Grey.seal_ = 32; *n*_Harbour.seal_ = 29).

In total, intestinal samples of 33 grey seals of both seas were investigated and showed an MP share of zero to 43 particles per individual (M ± SD = 7.91 ± 9.83). In comparison, a MP burden of zero to 81 MPs was determined in 30 harbour seals (M ± SD = 9.45 ± 16.81). No significant differences were revealed within the application of the *t*-test (*p*-value = 0.67). In addition, an intestinal sample of a grey seal with an unknown place of finding was processed, and five fibres and no fragments were determined. Harbour seals in the NS are more burdened than in the BS (M_NS_ = 7.67, M_BS_ = 14.13), whereas the intestinal samples of the grey seals showed a higher MP occurrence in the NS (M_NS_ = 8.94; M_BS_ = 6.79). The individual findings categorised by sea and species are given in Figure 1.

### 3.3. Influence of Age & Sex

In female seals, the mean MP presence was higher (M ± SD = 10.37 ± 18.7) compared to the occurrences in males (M ± SD = 7.44 ± 7.31). In addition, the four highest MP findings were found in females (a range of 32–81 MPs per individual). Nevertheless, no significant differences between the sexes (*p*-value = 0.45) were identified.

**Figure 2:**
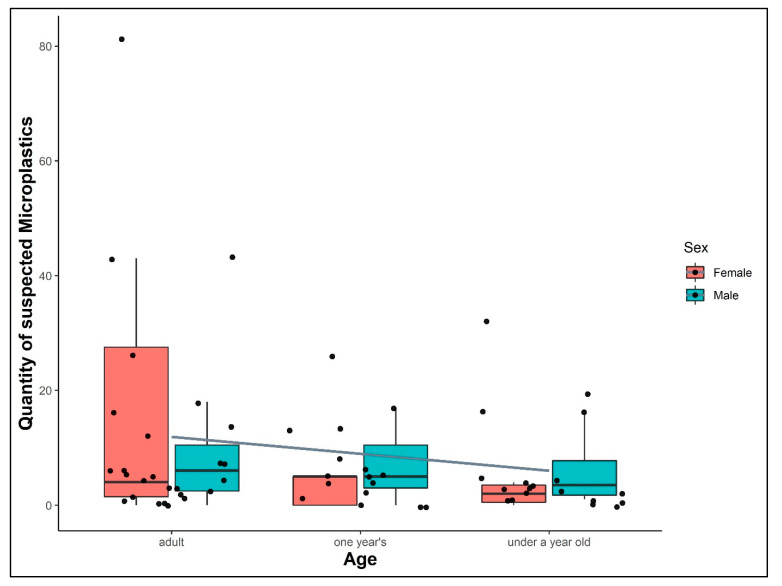
Quantity of suspected microplastics found in intestinal samples of different age groups and sexes of seals (harbour seals and grey seals) from German waters (North Sea and Baltic Sea; *n* = 62). The grey line is computed only by the mean MP findings out of the individuals per age group and elucidates the decreased quantity of MPs in younger specimens. The dataset is presented as boxplot behind the scatter plot for each category (sex or age), to visualise the distribution of MP findings in each group.

Moreover, the numbers of suspected MPs decrease from adults to individuals younger than a year (M_adult_ = 11.92, M_one year’s_ = 6.81, M_under a year’s_ = 6). In addition, the age (*p*-value = 0.29) seems to have more impact on the MP burden than the sex (*p*-value = 0.35). The grey line in Figure 2 is computed out of the mean MP findings per each age group and elucidates the lower MP occurrence in younger specimens.

### 3.4. Pathological Changes of the GIT

In total, 62 intestinal samples of harbour seals and grey seals were investigated for MP presence. Apart from one specimen, where only a stomach and no intestinal sample was obtainable for further analysis, the other six stomachs are comparable with intestinal samples from the same individual (Table 1).

#### 3.4.1. Stomach

MP presence was identified in all seven investigated stomachs (16 to 53 particles per individual; 28% fibres, 72% fragments). Two of those seven stomachs showed multifocal mural inflammatory lesions (a mild multifocal mural gastritis and a moderate to severe granulomatous eosinophilic gastritis). Furthermore, in both cases a mild parasite infestation of nematodes was found. Besides these lesions, those two specimens showed a medium range of MP findings (23 and 27 particles). In addition, no correlation between the number of parasites and the number of MPs in both, the stomach or in the intestine could be identified. In the remaining five stomachs, no inflammatory or degenerative changes were identified although three were infested with nematodes (16 to 53 MPs per specimen).

#### 3.4.2. Intestine

Within the 62 intestinal samples, a range of zero to 81 MP particles were identified. Nine out of these specimens were assumed to be unburdened by microplastics, since no particles remained after subtraction of considered secondary contamination. In addition, no or mild parasite infestation in these intestines were determined. The highest number of MPs was found in a harbour seal originating from the BS with a moderate acanthocephalan and a severe nematode infestation. In other 16 individuals, a mild to moderate enteritis with different disease patterns was identified. In three out of those, no parasite infestation was determined. Furthermore, out of all 62 intestines, nine showed activated Peyer‘s patch cells, in which the MP presence ranged between 0–5 found particles per specimen. In total, 42% of all suspected MP in the intestinal samples were categorised as fibres and the remaining 58% as fragments. Nevertheless, no statistically significant correlation between MP occurrence and inflammatory lesions or parasite infestations was identified, when comparing the findings in this study.

## 4. Discussion

This study was conducted to evaluate the MP burden in seals from German waters as well as gaining an overview of possible correlations between life history parameter and MP uptake. In addition, this is the first study dealing with an extended amount of specimen originating from seal species of the German NS and BS. Compared to information from the BS, research results of MP burden in species from the Northeast Atlantic area are available. Thus, in the following we will mainly focus on this region for discussing our results and to put it into the right context.

In total, 535 MP particles were found. To avoid an overestimation of the actual burden, 27 blanks were taken into account. Furthermore, Philipp et al. [16] already determined a true positive rate of 84% to 90% of MP identification if the described analysis is applied. In accordance, applying this method, 450–482 out of the 535 recognized MPs are reliable acknowledged as synthesised or artificial plastic particles (100–5000 µm), even though no polymer identification was conducted. However, the utilisation of a polymer identification tool such as µRaman spectroscopy is in general advisable, but during this study not necessarily needed, since the aim of this study was rather to identify tissue damage and after-effects in MP burdened seals.

However, to allow a further stabilised and detailed overview of a microplastic burden and their effects in marine mammals, it is in general recommended for future studies to investigate additional samples of the GIT (stomach and intestine) and a specific number of specimens being investigated exclusively on MP research, as it was already performed by different studies in the past [17,19,40]. In case of the investigation of the whole GIT on the occurrence of MPs it can be considered for future studies to analyse the samples firstly for MP presence and in further steps investigated on, e.g., parasite infestations or bacteriological analyses. Thus, a combination of different subject areas is highly recommended for future studies.

Since it is already considered that the MP occurrence in predatory marine mammals occurs exclusively through the uptake of prey species [8,9,17], it is not surprising that the MP load in several fish species is already confirmed [41,42]. In accordance with the approved uptake, the egestion of MP is evidenced, since some studies even indicated the presence of those particles in scat samples collected on different haul-out sites of seals [16,43,44]. Those studies focused on MPs with at least a size of 100 µm. Thus, the presence of those larger synthesised particles in the GIT of different marine mammal species is studied, albeit the fate of smaller ones is still questionable in general for mammal specimen [45]. The evaluation of the occurrence of even smaller particles is essential since it cannot be ruled out that those size classes might pass into the tissue. As already indicated by Nelms et al. [17] and Philipp et al. [16], the total numbers of found MP particles and the amount of fibres and fragments can differ between the GIT compartments and scats. This also agrees with the results presented here. The smaller amount of found fragments in scat samples [16] or in intestinal samples, in comparison to the findings in stomachs, supports previous mentioned studies on the potential retainment of MPs within the GIT tissue. It is reasonable to assume that aggregations of hard and indigestible particles, such as MPs, result in alterations of the intestinal or stomach tissue, such as enteritis or gastritis, as it is already indicated in invertebrates and mice [25,26].

### 4.1. North Sea & Baltic Sea

The MP burden in the investigated individuals originating from the BS (M ± SD = 9.45 ± 16.92) is higher than in those found in the NS (M ± SD = 8.29 ± 11.62), even no significant differences were identified (*p*-value = 0.78). Nevertheless, a larger sample size and an equal sample size out of both seas, and per species are recommended for future studies to further underline these results.

Since similar results were shown in previous studies focusing on harbour porpoises [14] or in fish from both seas [41,46], a significant difference is not highly expected. In contrast, studies investigating water or sediment samples of the NS or the BS showed a higher MP presence in the NS [47,48]. Since studies analysing MP presence in the BS from different media are still scarce, a comparison is constricted. Nevertheless, different models of MP distribution calculated, e.g., higher microfiber presence in the BS, comparing to the NS region [49]. However, the occurrence of MPs varied highly between the investigated media (e.g., stomach contents of different species, sea surface or sediments) and the focused objects (fragments, fibres, films, etc.) due to differences e.g., in buoyancy and the focused areas (e.g., point sources, influx, anthropogenic pressure) [17,50]. In accordance, only comparisons of samples of the same species or species with a similar diet or feeding behaviour, which are distributed in the same area, should be considered. The presented study adheres to this specification and thus gives first reliable evidence in the MP exposure on seals from the NS and BS.

### 4.2. Pathological Lesions

Some studies have already addressed MP occurrence and lesions in concerned GIT parts and indicated a risk of inflammatory lesions in the GIT tissue of free-ranging marine invertebrates or vertebrates (25). Moreover, laboratory studies focusing on mice or mammalian cells found further evidence for relations between MP presence and cell lesions in the GIT (26). Nevertheless, the knowledge of MP occurrence and resulting lesions in free-ranging mammals, especially in marine mammals, is lacking. Since it is not possible to feed marine mammals with plastic due to ethical reasons, the only option is to compare results from laboratory studies with invertebrates or laboratory species to gain first evidence.

In accordance, this study revealed that in 31.25% out of all displayed enteritis (*n* = 16), the etiology was more likely due to parasitic infection. Furthermore, 50% out of 16 individuals displayed a mild to moderate gastritis possibly due to parasitic infestation. In addition, the specimen with the highest MP burden of 81 particles also showed a severe parasite infestation by an acanthocephalan species in the intestine. However, no clear connection can be proven here since these acanthocephalan species are common endoparasites in seals from the NS and BS [19,33] and etiology of inflammation due to other causes is likely. Moreover, a similar study found first evidence of a possible correlation between parasite occurrence and MP presence [19]. Nevertheless, neither this latter mentioned, nor this here presented study indicated a significant positive relation between those parameters.

To further identify possible ramifications, changes of the Peyer’s patches were considered, since the uptake and transfer of MP via endocytosis is proven in different laboratory studies dealing with small mammalian species as reviewed by Wright & Kelly [51]. Nevertheless, no coherence was found between MP presence and changes in Peyer‘s patches in the intestines of the investigated seals. In addition, the results of this study give no evidence for relations between MP occurrence (>100 µm) and inflammatory responses in the tissue, so far. In addition, some studies dealing with invertebrate species already identified alterations in the GIT if MP or nanoplastic are present [52,53]. Furthermore, the exposure to MP (5 µm and 20 µm in size) leads to accumulation and inflammation in liver, spleen and guts as it was already reported in mice [54].

However, specific investigations on the correlation of intestinal lesions and the occurrence of MPs need to be conducted, also including other etiologies for lesions, to further understand the impact of MP on the health of marine mammals.

In case of a high occurrence of both in a number of individuals, it is advisable to identify the present parasites on a species level to determine if one specific species enhances MP presence. Moreover, a detailed histological and pathological investigation in terms of MPs in the burdened GIT tissue needs to be considered, to (1) detect MP and lesions in the tissue itself and (2) to determine the likelihood of the specific MP found, triggering the indicated lesion. However, all recent applied protocols focusing on MP burden in marine mammal GITs are dealing only with the gut content [17,19,40], since identification tools such as staining or spectroscopic approaches are not yet applicable to the tissue itself.

Furthermore, the main part of the investigated carcasses in this study (37 out of 63), were in a state of starting or advanced decomposition (Code 3–5 after Ijsseldijk et al. [35]), which limited a reliable histological judgement in these carcasses. Moreover, we would recommend examining other organs then the GIT, to amplify the knowledge of effects of MP and nanoplastic in burdened marine mammals.

### 4.3. Differences of MP Occurrence in Age & Sex

This is the first study dealing with MP occurrence and life history parameter of seals from the North Sea and Baltic Sea found on the German coastlines. Since no significant differences between sexes or ages could be identified, this study coincides with an already published research of no accumulation of MPs within the intestine of marine mammals [9]. Nevertheless, a higher MP burden was indicated in females and adult specimen. One possible hypothesis to explain this finding could likely be a higher uptake rate of prey species in adults than in younger individuals, and the increased energy needs for gestation and lactation [55]. Moreover, feeding quantity, the feeding behaviour, or favoured prey and foraging area differs between sexes, ages and species, as well as individual seals in general [10,11,12,13]. Hence, the exposure of MP and thus the presence in the GIT prior excretion can vary. First evidence of the effect of favoured prey species correlating with MP presence was already identified in harbour porpoises occurring in the same study area [14], but also in grey seals around Great Britain [56]. According to a small sample size of suspected bycaught individuals (*n* = 5), and an inconsistent distribution of MP presence (zero to 19 MPs per individual), no correlation between a bycaught specimen and MP presence was identified, as it was evidenced in harbour porpoises [14].

Moreover, all findings of marine litter in a size which is visible by the naked eye are recorded for the here examined seal carcasses as it is already published in a previous study [2]. In accordance, a rubber bait was found next to two stones in the stomach of a harbour seal originating from the NS in 2014. Unfortunately, the stomach content of this individual was not investigated for MP analysis. Nevertheless, the intestinal sample showed a MP occurrence of only three particles. However, the ingestion of fishing gear in marine mammals is not uncommon [2,18], and a degradation of synthesized objects in the GIT due to peristalsis and gastric acid is conceivable [3]. Nevertheless, a link between the ingestion of a plastic object and a high burden of MP in the GIT is not indicated in this study, though this may be based on the low sample size.

## 5. Conclusions

Studies investigating MP presence in seals from the BS are still missing. Thus, this study gives first evidence on the addressed burden in harbour seals and grey seals occurring in German waters (NS and BS). Nevertheless, no significant difference was identified between those two sympatric species. This indicates that the MP exposure for each species is at a similar level. In accordance, comparable surveys between the NS and BS, and the two seal species are lacking, so this study was needed to highlight differences in the occurrence of MP and its possible effects in harbour seals and grey seals from those areas. Similar to previous studies, the MP presence in marine mammal specimen from the BS were higher, if compared with those originating from the NS. Consequently, a higher MP exposure in the BS to marine mammals, especially for seals as indicated, is assumed. Nevertheless, no significant differences in microplastic loads between sexes or ages were revealed, which underlines the hypothesis of the egestion of MP particles (100–5000 µm). However, an accumulation of indigestible particles in the stomach compartment is considerable since the higher numbers of found MP within this investigation and previous studies indicate a restraint. Since information on undersized particles is still missing, further studies should focus on particles smaller than 100 µm, and their fates and pathways in vivo.

## Figures and Tables

**Table 1:** Overview of microplastic occurrence in stomach contents and intestinal samples from seals found in the German North Sea and Baltic Sea in 2019.

Species	Microplastic Findings		Share of Fibres		Share of Fragments	
	Stomach	Intestine	Stomach	Intestine	Stomach	Intestine
Grey seal (BS)	37	13	5	4	32	9
Harbour seal (BS)	47	5	10	5	37	0
Harbour seal (NS)	28	8	11	4	17	4
Harbour seal (NS)	53	1	17	1	36	0
Harbour seal (NS)	25	2	5	2	20	0
Harbour seal (NS)	16	32	5	28	11	4
Harbour seal (NS)	23	NA	12	NA	11	NA

## Data Availability

The data presented in this study are available on request from the corresponding author.
